# Clinical and epidemiological differences in staphylococcal osteoarticular infections: insights for developing hospital-based infection control interventions

**DOI:** 10.1007/s00590-025-04184-w

**Published:** 2025-02-08

**Authors:** João Figueiredo, Jorge Lindo, Catarina Chaves, Célia Nogueira

**Affiliations:** 1 FMUC—Faculty of Medicine, University Coimbra, Coimbra, Portugal; 2 Centro Hospitalar e Universitário de Coimbra, Coimbra, Portugal; 3 CNC-UC - Centre for Neuroscience and Cell Biology, University Coimbra, Coimbra, Portugal; 4 CiBB - Centre for Innovative Biomedicine and Biotechnology, University of Coimbra, Coimbra, Portugal; 5 Microbiology Laboratory, Pathology Unit, Centro Hospitalar e Universitário de Coimbra, Portugal, Coimbra, Portugal

**Keywords:** Osteoarticular infections, *Staphylococcus aureus*, *Coagulase-negative Staphylococcus*, Epidemiology, Antimicrobial resistance

## Abstract

**Purpose:**

Osteoarticular infections (OAI) are serious clinical conditions with *Staphylococcus aureus* and Coagulase-negative *Staphylococcus* (CoNS) responsible for up to two-thirds of cases. This work aimed to compare the epidemiological, clinical, and microbiological characteristics of OAI caused by *S. aureus versus* CoNS to aid in clinical management and infection control strategies.

**Methods:**

A single-centre retrospective study was performed at the Centro Hospitalar e Universitário de Coimbra for the period of January 2011 to December 2021. A total of 458 cases of OAI were gathered. Data was retrieved from medical records and statistical analysis was performed with SPSS.

**Results:**

*S. aureus* accounted for 60.7% of infections, followed by *S. epidermidis* (29.9%)*.* Independent risk factors for *S. aureus* infections included being male (*p* < 0.001; OR = 0.47) and a history of osteomyelitis (*p* < 0.001; OR = 0.18). In contrast, CoNS infections were associated with older age (*p* = 0.018), carrying a prosthetic device (*p* < 0.001; OR = 2.92), and a prior periprosthetic infection (*p* = 0.023; OR = 1.86). Both groups exhibited significant antimicrobial resistance, with CoNS showing greater resistance to gentamicin, linezolid, teicoplanin and trimethoprim-sulfamethoxazole, while *S. aureus* was more commonly resistant to clindamycin.

**Conclusion:**

Our findings show the distinct characteristics of OAI caused by *S. aureus* and CoNS, highlighting the need for targeted risk factor management and tailored empiric antibiotic therapy to reduce incidence and improve outcomes.

**Supplementary Information:**

The online version contains supplementary material available at 10.1007/s00590-025-04184-w.

## Introduction

Osteoarticular infections (OAI) prevalence is increasing with potentially severe consequences for the skeletal development and function, as well as for the patients’ quality of life [1]. OAI can generally be categorized into two groups: osteomyelitis and septic arthritis. The first one refers to the infection of the bone, while the latter one involves the infection of joints. Clinically, OAI may present with localized pain, decreased joint mobility, inflammatory signs of the skin, fever, among others [2, 3]. Clinical findings alone are insufficient to establish the diagnosis. Thus, imaging studies, along with microbiological analysis, are crucial diagnostic tools that enable clinicians to provide the most effective treatment options [2, 3].

Several risk factors have been identified for the development of OAI, namely diabetes, ageing, osteoarthritis, rheumatoid arthritis, previous bone or joint surgery, dermatologic infections, haemodialysis, intravenous drug use, peripheral vascular disease, immunosuppression, among others [2, 4–6]. However, the results are not consistent across studies. Notably, advances in modern technology and an ageing population have increased the use of orthopaedic devices like joint prostheses and osteosynthesis hardware [1]. These devices are key risk factors for OAI due to their potential to support biofilm formation [2].

*Staphylococcus* spp., *Streptococcus* spp., and aerobic Gram-negative bacilli are frequent pathogens of OAI, with *Staphylococcus aureus* being the most prevalent [2, 7]. In fact, *S. aureus* can invade, colonize and destroy the bone through multiple mechanisms [7]. Together with Coagulase-negative *Staphylococcus* (CoNS), such as *S. epidermidis, S. lugdunensis, S. hominis, S. haemolyticus and S. warneri, S. aureus* is responsible for up to two- thirds of all OAI [7–9].

*Staphylococcus*-related OAI present a significant clinical challenge, particularly regarding the optimal choice of antibiotics and treatment duration. [2, 10, 11]. Generally, empirical antibiotic therapy should be initiated promptly, guided by local antimicrobial resistance patterns or, if available, by the resistance profiles of pathogens isolated from specimens collected from sites (such as blood) other than the primary infection site in the same patient [2, 10].

There has been a continuous decline in susceptibility to many antibiotics used for *S. aureus* and CoNS [8, 12]. Methicillin-resistant *S. aureus* (MRSA) prevalence has been rising and is found not only in healthcare-associated infections but also in community-acquired infections. [6, 12]. Additionally, CoNS are progressively more resistant to single antibiotic therapies, and often exhibit multidrug resistant [8]. This is a significant concern because CoNS are thought to be a reservoir of genetic elements that confer antibiotic resistance, which can be transferred to *S. aureus* [8].

Given the clinical importance of *Staphylococcus*-related OAI, we aimed to compare the epidemiological and clinical characteristics of patients and the antibiotic susceptibility profiles of *S. aureus*-induced OAI with CoNS-induced OAI. Our goal was to identify risk factors for these infections, provide locally tailored recommendations to optimize empirical antibiotic therapy, and develop hospital-based infection control interventions applicable to similar healthcare settings.

## Materials and methods

### Settings and study design

The present work is a single-centre, observational retrospective study conducted over a ten-year period (January 2011 to December 2021) at a tertiary university hospital centre. Institutional Ethics Committee approved the study (CHUC-UID.CEC.OBS.SF.208/2022), and informed consent was not required because the data was anonymized with a random patient identification number.

The database comprised 458 adult patients from Centro Hospitalar e Universitário de Coimbra (CHUC) who had samples of synovial fluid, wound aspirate, abscesses, or biopsies with positives cultures for *S. aureus* or CoNS*.* Demographic and clinical data were retrieved retrospectively from the electronic medical records of the patients, namely sex, age, site of infection, pre-existing diagnoses, antibiotic susceptibility testing, and antibiotic therapy.

To avoid data clustering, we excluded recurrent cases, as well as paediatric cases and pregnant women.

### Microbiological analysis

Bacterial identification was performed using the *Vitek® MS* (bioMérieux, France) and *Bruker®MS* (Siemens, USA) automated systems. Antimicrobial susceptibilities were determined with the *Vitek®2* (bioMérieux®, France) and *MicroScan Walkaway®* (Beckman Coulter, USA) automated systems, according to European Committee on Antimicrobial Susceptibility Testing guidelines (www.eucast.org).

### Statistical analysis

Statistical analysis was performed using *IBM SPSS Statistics* for Windows, version 27.0 (SPSS Inc, Chicago, IL, USA). The descriptive analysis was carried out, with quantitative variables expressed as mean ± standard deviation and qualitative variables as frequencies. The inferential analysis was performed using the chi-square (χ2) test and the Fisher´s exact test, as appropriate, followed by binary logistic regression. The χ2 test was applied when the *Cochran* rules were fulfilled. The *Mann–Whitney* and *Kruskal–Wallis* tests were employed to compare nominal variables with quantitative ones. A test was considered significant if *p* ≤ 0.05. Results were presented using graphics and tables created in *MS® Excel®*

## Results

### Characteristics and temporal distribution of *Staphylococcus* spp. in the population

A total of 458 episodes of OAI caused by Gram-positive cocci were reviewed. Among these, 62% (284) were males and 38% (174) were females, resulting in a male to female ratio of 1.63. Patient’s age ranged from 19 to 96 years, with a median age of 65.5 (± 16.1) years. Table 1 shows the distribution of *Staphylococcus* spp. according to age group and sex. The number of OAI increased up to the age group of 60 to 79 years. In fact, most infections occurred in individuals over 60 years old compared to younger individuals (64.4% *vs.* 35.6%).

**Table 1:** Distribution of *Staphylococcus* spp. according to sex and age

	19–39 years			40–59 years			60–79 years			> 80 years			Global		
	F^a^	M^a^	Total^b^	F^a^	M^a^	Total^b^	F^a^	M^a^	Total^b^	F^a^	M^a^	Total^b^	F^a^	M^a^	Total^b^
Microorganisms	*n* = 14	*n* = 32	*n* = 46	*n* = 33	*n* = 84	*n* = 117	*n* = 92	*n* = 128	*n* = 220	*n* = 35	*n* = 40	*n* = 75	*n* = 174	*n* = 284	*n* = 458
*Staphylococcus aureus*	21%	79%	29	29%	71%	83	34%	66%	119	34%	66%	47	31%	69%	278
*Staphylococcus epidermidis*	50%	50%	16	28%	72%	25	53%	47%	81	67%	33%	15	50%	50%	137
*Staphylococcus lugdunensis*	0%	100%	1	17%	83%	6	40%	60%	10	71%	29%	7	42%	58%	24
*Staphylococcus warneri*	–	–	0	50%	50%	2	33%	67%	3	100%	0%	1	50%	50%	6
*Staphylococcus haemolyticus*	–	–	0	0%	100%	1	100%	0%	2	75%	25%	4	71%	29%	7
*Staphylococcus hominis*	–	–	0	–	–	0	40%	60%	5	0%	100%	1	33%	67%	6
*Coagulase-negative Staphylococcus*	47%	53%	17	26%	74%	34	51%	49%	101	68%	32%	28	49%	51%	180
Total	30%	70%	46	28%	72%	117	42%	58%	220	47%	53%	75	174	284	458

^a^Values are expressed in relative frequency (%). Female (F); Male (M)

^b^Values are expressed in absolute frequency (n)

Among the isolates, *S. aureus* was the most common organism identified, accounting for 60.7% (n = 278). The remaining samples consisted of CoNS, namely *S. epidermidis* (29.9%, *n* = 137), *S. lugdunensis* (5.2%), *S. haemolyticus* (1.5%), *S. warneri* (1.3%), and *S. hominis* (1.3%). Across all age groups, *S. aureus* was the most frequently isolated pathogen*,* followed by *S. epidermidis* and then *S. lugdunensis*. CoNS were 1.70 times more likely to affect patients older than 60 years compared to *S. aureus* (*p* = 0.009; OR = 1.70; 95% CI = 1.14–2.55).

Regarding the distribution of *Staphylococcus* species by patient gender, a higher frequency was observed in males for almost all pathogens, with the exceptions of *S. epidermidis* and *S. warneri* which had equal frequencies between the sexes, and *S. haemolyticus* that was more common in females. CoNS were 2.14 times less likely to affect males than *S. aureus* (*p* < 0.001; OR = 0.47; 95% CI = 0.32–0.69).

**Fig. 1:**
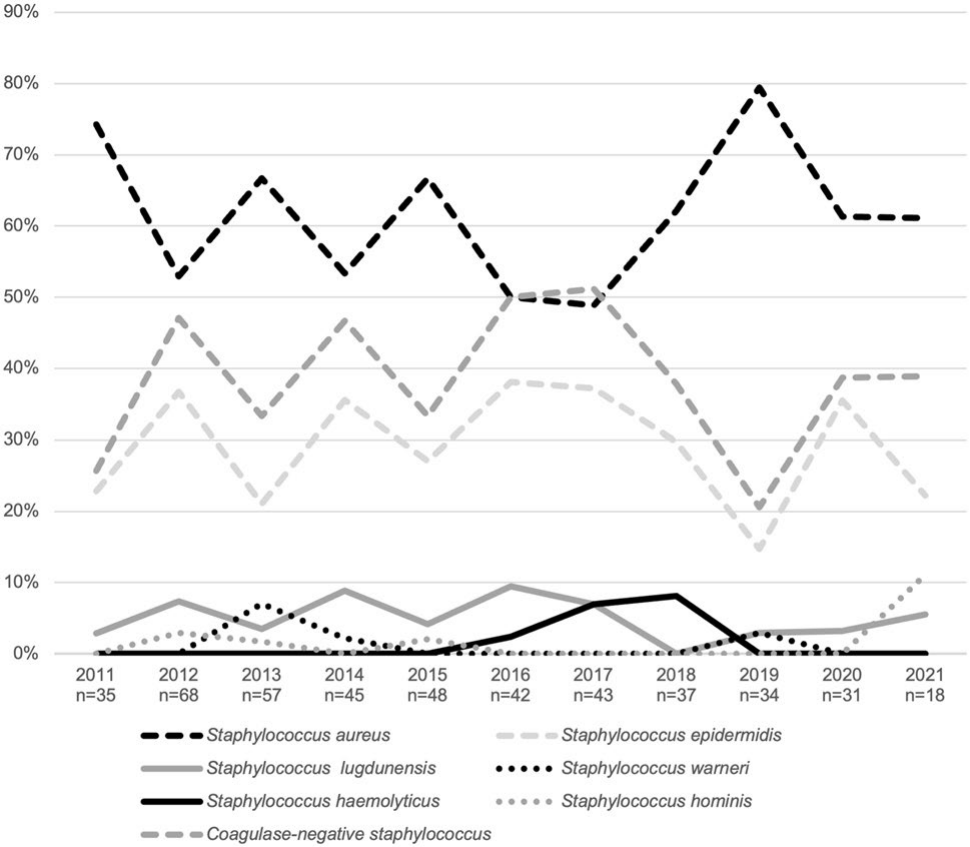
Frequency of pathogens over the ten-year period (relative frequency in %)

The distribution of isolated staphylococci exhibited slight fluctuations over the 10-year period (Fig. 1), with *S. aureus* being the most frequent isolated species, followed by *S. epidermidis*. The frequency of *S. aureus* was consistently equal to or higher than the combined frequency of all the CoNS together, except in 2017, when CoNS were isolated in 51.2% and *S. aureus* in 48.8%. Overall, there were globally statistically significant differences in the frequencies of CoNS (mean = 39.3%) compared to *S. aureus* (mean = 60.7%), across the years (*p* = 0.016).

### Clinical characteristics and comorbidities associated with *Staphylococcus* spp. infection

Table 2 summarizes the patient’s clinical characteristics. Cardiovascular disorders (35.4%; *n* = 162) and oncological conditions (15.2%; *n* = 71) were present in a significant proportion of participants. Among these, the most common comorbidities were diabetes and dyslipidaemia. Furthermore, many patients presented a history of recent surgery (39.3%; *n* = 180) and of periprosthetic infections (13.8%; *n* = 63) prior to the considered episode of infection. A notable number of patients also had a history of previous trauma (12%; *n* = 55), carried a joint prosthetic device (57.6%; *n* = 264) or had osteosynthesis material (10.7%; *n* = 49).

**Table 2:** Patient`s characteristics and comorbidities distributed by the main causative agents

	Variable	Total (*n* = 458)^a^	*S. aureus* (*n* = 278)^a^	CoNS (*n* = 180)^a^	*p*	OR^b^
Demographics	Age (years)	62.8 ± 16.1	61.6 ± 16.3	64.7 ± 15.6	0.018	–
	Sex (male)	284 (62.0%)	192 (69.1%)	92 (51.1%)	< 0.001	0.468
Addictions	Smoking	14 (3.1%)	12 (4.3%)	2 (1.1%)	0.056	
	Alcohol abuse	10 (2.2%)	6 (2.2%)	4 (2.2%)	1	–
	Intravenous drug use	5 (1.1%)	4 (1.4%)	1 (0.6%)	0.653	–
Therapies	Corticotherapy	4 (0.9%)	3 (1.1%)	1 (0.6%)	1	–
	Immunosuppressive therapy	16 (3.5%)	7 (2.5%)	9 (5.0%)	0.158	–
	Chemotherapy/Radiotherapy	13 (2.8%)	5 (1.8%)	8 (4.4%)	0.096	–
Orthopaedics	Osteomyelitis	43 (9.4%)	38 (13.7%)	5 (2.8%)	< 0.001	0.180
	Septic arthritis	17 (3.7%)	10 (3.6%)	7 (3.9%)	0.872	–
	Gout	25 (5.5%)	18 (6.5%)	7 (3.9%)	0.234	–
	Rheumatoid arthritis	17 (3.7%)	9 (3.2%)	8 (4.4%)	0.505	–
	Lupus	8 (1.7%)	6 (2.2%)	2 (1.1%)	0.489	–
	Seronegative arthritis	41 (9.0%)	29 (10.4%)	12 (6.7%)	0.168	–
	Osteoarthrosis	15 (3.3%)	10 (3.6%)	5 (2.8%)	0.630	–
	Osteoporosis	16 (3.5%)	7 (2.5%)	9 (5.0%)	0.158	–
	Previous bone fracture	76 (16.6%)	48 (17.3%)	28 (15.6%)	0.631	–
	Amputation	12 (2.6%)	8 (2.9%)	4 (2.2%)	0.772	–
	Joint prosthetic device	264 (57.6%)	133 (47.8%)	131 (72.8%)	< 0.001	2.915
	Previous periprosthetic infection	63 (13.8%)	30 (10.8%)	33 (18.3%)	0.022	1.856
	Osteosynthesis material	49 (10.7%)	32 (11.5%)	17 (9.4%)	0.485	–
Skin and mucous membranes	Skin and soft tissue infections	40 (8.7%)	30 (10.8%)	10 (5.6%)	0.053	–
	Trauma	55 (12.0%)	39 (14.0%)	16 (8.9%)	0.098	–
	Wounds/incisions	14 (3.1%)	11 (4.0%)	3 (1.7%)	0.266	–
	Fistula	6 (1.3%)	5 (1.8%)	1 (0.6%)	0.411	–
Endocrine	Diabetes mellitus	72 (15.7%)	49 (17.6%)	23 (12.8%)	0.164	–
	Dyslipidaemia	68 (14.8%)	35 (12.6%)	33 (18.3%)	0.091	–
	Thyroid disease	17 (3.7%)	13 (4.7%)	4 (2.2%)	0.212	–
	Pancreatic disease	4 (0.9%)	3 (1.1%)	1 (0.6%)	1	–
	Obesity	25 (5.5%)	13 (4.7%)	12 (6.7%)	0.360	–
Invasive procedures	Recent surgery	180 (39.3%)	108 (38.8%)	72 (40.0%)	0.805	–
	Endovascular prosthetic devices	7 (1.5%)	4 (1.4%)	3 (1.7%)	1	–
	Other invasive medical procedures	6 (1.3%)	5 (1.8%)	1 (0.6%)	0.411	–
Nephrology	Haemodialysis	2 (0.4%)	0 (0.0%)	2 (1.1%)	0.154	–
	Chronic kidney disease	26 (5.7%)	19 (6.8%)	7 (3.9%)	0.183	–
Other comorbidities	Cardiovascular disease	162 (35.4%)	97 (34.9%)	65 (36.1%)	0.790	–
	Oncological disorders	71 (15.5%)	38 (13.7%)	33 (18.3%)	0.178	–
	Hepatic disease	19 (4.1%)	13 (4.7%)	6 (3.3%)	0.481	–
	Sexually transmitted diseases	12 (2.6%)	10 (3.6%)	2 (1.1%)	0.138	–
	Other organs infection	9 (2.0%)	7 (2.5%)	2 (1.1%)	0.493	–

^a^Data are expressed as n (%) or mean ± SD

^b^OR, *Odds-Ratio*. Presented only when the result was significant

Carrying a prosthetic device (*p* < 0.001), having a prior periprosthetic infection (*p* = 0.022) or a history of osteomyelitis (*p* < 0.001) and being male (*p* < 0.001) were identified as significant risk factors for OAI caused by specific pathogens. Specifically, having a prosthetic device raised the risk of developing an OAI by CoNS by 2.92 times (*p* < 0.001; OR = 2.92; 95% CI = 1.95–4.37)*.* A history of an infected prosthetic device was associated with a 1.86-fold increase (*p* = 0.023; OR = 1.86; 95% CI = 1.09–3.17) in the risk of CoNS infection*.* Conversely, a history of osteomyelitis increased the risk of developing an OAI due to *S. aureus* by 5.56 times (*p* < 0.001; OR = 0.18; 95% CI = 0.07–0.47). Being male was associated with a 2.14-fold higher risk (*p* < 0.001; OR = 0.47; 95% CI = 0.32–0.69) of developing an infection caused by *S. aureus.* Two additional findings, although not statistically significant, suggested potential associations: smoking (*p* = 0.056) and the occurrence of skin and soft tissue infections (*p* = 0.053) may be linked to an increased likelihood of *S. aureus* OAI.

For the remaining variables, there were no differences between the groups (*p* > 0.05).

### *Staphylococcus* spp. antibiotic resistance pattern

**Fig. 2:**
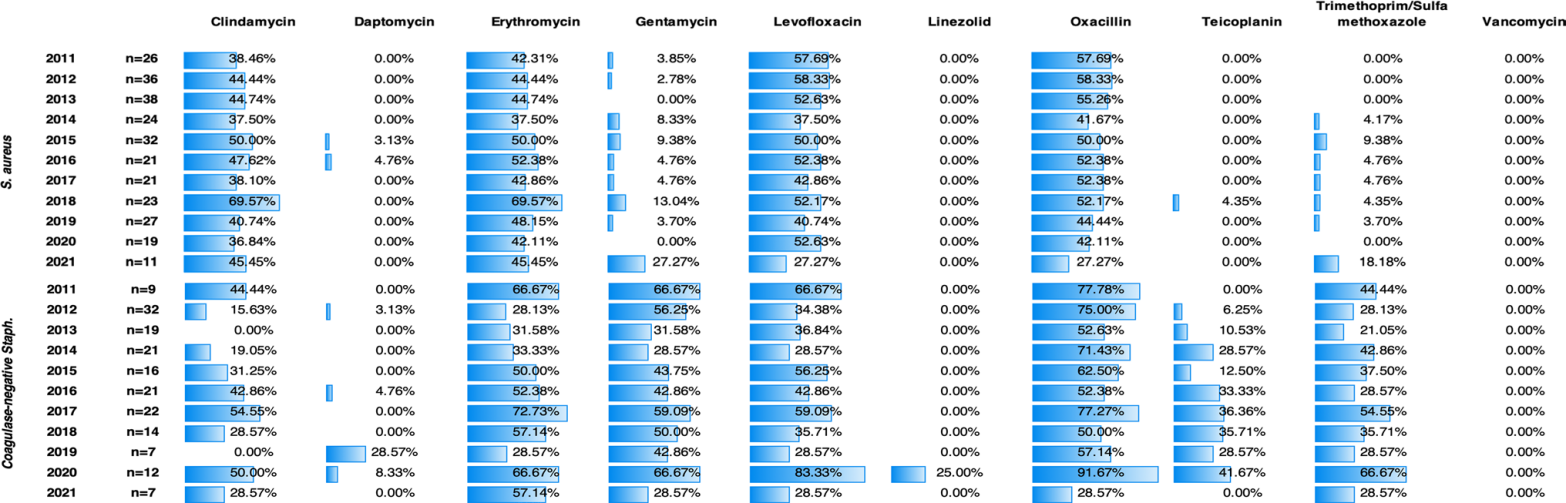
Antibiotic resistance (%) of *S. aureus* and CoNS, 2011–2021

The antimicrobial resistance data for isolated staphylococci, from 2011 to 2021, are shown in Fig. 2. Throughout the study period, *S. aureus* showed high levels of resistance to clindamycin, erythromycin, levofloxacin, and oxacillin. Clindamycin and erythromycin followed a similar trend, with relatively stable susceptibility profiles over times, except in 2018, when a significant increase in resistant strains was observed, reaching approximately 70%. Regarding levofloxacin and oxacillin, resistance was generally observed in about half of the *S. aureus* isolates. However, in 2021, there was a notable reduction in resistance, with rates dropping to 27.27%, nearly half of the values from the previous year. Resistance to daptomycin and teicoplanin was consistently below 5%, with these antibiotics showing 100% sensitivity in most years. As for gentamicin and trimethoprim-sulfamethoxazole, resistance levels were generally low, typically under 10%. Nevertheless, in 2021, there was an increase in resistance, with rates rising to 27.27% for gentamicin and 18.18% for trimethoprim-sulfamethoxazole. No resistance to vancomycin and linezolid was seen.

Concerning the susceptibility profile of CoNS, *S. epidermidis* made the largest contribution, representing 76% of all CoNS isolates. As shown in Fig. 2, CoNS displayed a more pronounced resistance pattern compared to *S. aureus*. High levels of resistance were observed for erythromycin, gentamicin, levofloxacin, oxacillin, and trimethoprim-sulfamethoxazole. Methicillin/oxacillin resistance in CoNS ranged from 28.57% to 91.67%, with an average of 65.56%. Resistance to clindamycin and teicoplanin was also high. While clindamycin resistance exhibited an irregular pattern, teicoplanin resistance remained consistently high during the second half of the study, except for a sharp decrease to 0% in 2021. Vancomycin and linezolid were the most effective antibiotics against CoNS, with no resistant strains reported, except for linezolid in 2020, when resistance reached 25%, primarily due to its inefficacy against *S. epidermidis* (Online Resource 1). Most CoNS remained susceptible to daptomycin, apart from 2019, when 28.57% of isolates demonstrated resistance.

As expected, the antimicrobial resistance pattern of *S. aureus* was statistically different from that of CoNS (*p* < 0.05). CoNS isolates were more frequently resistant to gentamicin (*p* < 0.001; OR = 15.82; 95% CI = 8.80–28.42), linezolid (*p* = 0.03), oxacillin (*p* = 0.001; OR = 1.96; 95% CI = 1.32–2.90), teicoplanin (*p* < 0.001; OR = 83.44; 95% CI = 11.34–614.04) and trimethoprim-sulfamethoxazole (*p* < 0.001; OR = 16.32; 95% CI = 8.10–32.89) compared to *S. aureus*. On the opposite, *S. aureus* was 1.85 times more likely to be resistant to clindamycin than CoNS (*p* = 0.03; OR = 1.85; 95% CI = 1.23–2.77).

## Discussion

We conducted a ten-year (2011–2021) retrospective study at CHUC to investigate how OAI caused by *S. aureus* differ from that caused by CoNS, identify the associated risk factors, and assess the evolution of antibiotic resistance in these pathogens. The goal was to optimize empirical therapy and prophylaxis to better align with the evolving epidemiological landscape and develop targeted infection control interventions.

The *Staphylococcus* genus includes the most frequent etiological agents of OAI, accounting for approximately 75% of osteomyelitis [13] and around 50% of septic arthritis [6–11]. *S. aureus* is considered the most significant pathogen, but CoNS also play a substantial role [9, 13–16]. This is reflected in the present study, since *S. aureus* was the most prevalent microorganism, followed by *S. epidermidis* [17]. Overall, during the period under analysis, *S. aureus* was more prevalent than all CoNS combined, consistent with findings in the literature [6, 7, 11, 18]. However, other studies have found CoNS to be more frequently isolated than *S. aureus* [19–22].

Our data revealed that OAI were more frequent in males (62%) as has previously been reported in other Portuguese studies [11, 23] and internationally [1, 6, 9, 16, 24, and 25]. Male gender has been identified as a risk factor for *S. aureus* carriage [26], which may explain the higher risk of *S. aureus* infections in men. Previous studies highlighted an association between OAI due to CoNS and older patients, in contrast with OAI caused by *S. aureus* [6, 24, and 27]. Our findings support these reports, since CoNS were more likely to affect patients over 60 years of age compared to *S. aureus*.

*S. epidermidis* is well known for causing foreign body-related infection [8, 13], as it adheres to implants via adhesins [7] and forms biofilms [8]. Our results support that the risk of developing OAI related to orthopaedic implants is higher with CoNS. Specifically, patients with prosthetic devices or a history of periprosthetic infection [11] were found to have a higher risk of developing OAIs by CoNS than by *S. aureus*. This association was not observed for other conditions related to indwelling devices, such as haemodialysis and other invasive procedures due to the small number of cases in our study, which limited us to draw definitive conclusions.

*S. aureus* and *S. epidermidis* are responsible for most cases of osteomyelitis, as they colonize many asymptomatic individuals. Their ability to form small-colony variants [28] and biofilms enables them to persist, reinfect, and evade antibiotics, while spreading throughout the bone [13]. According to our study, there is a higher risk of infection by *S. aureus* over CoNS in patients with a history of osteomyelitis. Similar findings were observed in a cohort of patients with native septic arthritis, which showed that infections due to *S. aureus* were more often associated with the simultaneous occurrence of spinal osteomyelitis than those caused by non-*S. aureus* pathogens [6].

In our study, we found a higher prevalence of smoking among individuals infected with *S. aureus*, although this association was not statistically significant, perhaps due to the small number of cases. Previous investigations reported that smoking exposure can increase biofilm formation and enhance the binding of *S. aureus* to fibronectin [29], which is relevant for the invasion and colonization of host cells. Moreover Gobao et al. (2020) [6], showed an association between smoking and septic arthritis caused by MRSA *vs.* methicillin-susceptible *Staphylococcus aureus*. Rodriguez-Merchan and Delgado-Martinez (2022) [30] also noted that smoking is linked to higher incidences of periprosthetic joint infections. In this context, we recommend nasal screening for *S. aureus* carriage and implementing decolonization procedures among smokers with additional risk factors (e.g., male sex, previous osteomyelitis) who will undergo risk procedures (such as orthopaedic ones), to potentially prevent OAI. Similarly, we found a marginal association between dermatologic infections and *S. aureus* OAI. Notably, *S. aureus* is also the leading cause of skin and soft tissue infections which may explain this association [10]. Collectively, these findings may substantiate our results, indicating that smoking and the presence of dermatologic infections could be emerging risk factors for OAI caused by *S. aureus*.

Intravenous drug use has been identified by other studies [1] as a risk factor for OAI, in particular those involving *S. aureus* [6]. Nevertheless, our results did not support this association, likely due to the small number of intravenous drug users in our database. It is worth noting that of the five intravenous drug users, four were infected with *S. aureus.*

The management of OAI depends significantly on the effectiveness of perioperative antimicrobial therapy, whether it is administered with a curative intent or for preventive purposes. The selection of the antimicrobial drug is guided by established good practice guidelines and tailored to the implementation of protocols based on local epidemiology [19].

Regarding staphylococci, particular attention is given to resistance to methicillin/oxacillin, which is mediated by *mecA* gene [31]. In Portugal, the methicillin-resistance rate of *S. aureus* was 29.7% in 2020, exceeding the European average (16.7%), and ranking as the fourth highest among the 29 other European countries. However, this rate is significantly lower than the 54% that occurred in 2012 [32]. In Europe, a decline in MRSA incidence was also reported with rates decreasing from 18.4% in 2017 to 15.2% in 2022 [33]. In our study the rate of MRSA (50.36%) is very high when compared to the aforementioned data.

Nearly all CoNS clinical isolates are known to display methicillin-resistance [34]. In our study, CoNS were methicillin resistant in an average of 65.56% of cases. This prevalence is higher than the 45% reported by Titécat et al. (2013) [19], but lower than the 85% reported by Hellmark et al. (2009) [35] and the 76.4% reported by Putnam et al. (2010) [36]. A continuous decline in the susceptibility to many commonly used antibiotics has been observed for CoNS, particularly for oxacillin, clindamycin, erythromycin and gentamicin [8]. Similarly, we reported high rates of resistance to clindamycin, erythromycin, gentamicin, levofloxacin and trimethoprim-sulfamethoxazole. Throughout the examined period, there was also a noteworthy increase in CoNS resistance to teicoplanin [19, 37], from 0% in 2011 to 41.67% in 2020. While this increase aligns with literature reports [19, 37], the rates in our study were higher than those typically documented. In fact, some studies have shown that teicoplanin resistant CoNS are more common than vancomycin resistant strains, making vancomycin the preferred treatment for CoNS infections, since the therapeutic options are limited due to methicillin-resistance [8]. Resistance to teicoplanin should be carefully monitored, and larger population-based studies are needed to guide empirical antibiotic therapy with regard to this result. CoNS are a natural reservoir of genetic elements conferring antibiotic resistance, with the potential to be transferred to clinically significant staphylococcal species, such as *S. aureus* [8, 13, and 38]. Therefore, it is crucial to exercise special caution, particularly with CoNS, by closely monitoring the evolution of their resistance pattern, at least on a local level.

Regarding *S. aureus*, this study presents evidence of resistance to several antibiotics; on average 44.96% were resistant to clindamycin, 47.12% to erythromycin and 51.08% to levofloxacin. However, no vancomycin resistance was detected in our study, indicating that this antibiotic remains effective as an empirical treatment for OAI caused by staphylococci.

This study has limitations that should be acknowledged. Firstly, its observational and retrospective design introduces inherent biases in data collection. Secondly, as a single-centre study, the generalizability of our findings may be limited to other settings. Also, resolution of previous infections was considered based on clinical data, which may limit the interpretation of recurrence patterns.

In conclusion, OAI caused by *S. aureus* and CoNS exhibit distinct clinical and epidemiological characteristics. Antibiotic resistance remains a worrisome problem, especially with CoNS.

Recognising and addressing risk factors is essential for the successful implementation of preventive measures aimed at reducing the incidence of OAI and their associated morbidity, mortality, and costs. Effective antimicrobial stewardship can enhance patient safety, optimize resource utilization, and mitigate the emergence of resistance. Individuals who meet risk criteria should be identified upon admission and closely monitored to ensure that empirical antibiotic treatment is promptly initiated based on identified resistance patterns.

## Supplementary Information

Below is the link to the electronic supplementary material.

Supplementary file1 (TIFF 145779 KB)

## Data Availability

No datasets were generated or analysed during the current study.
